# Characterization and Complete Sequence of Lactonase Enzyme from *Bacillus weihenstephanensis* Isolate P65 with Potential Activity against Acyl Homoserine Lactone Signal Molecules

**DOI:** 10.1155/2013/192589

**Published:** 2013-07-09

**Authors:** Masarra Mohammed Sakr, Khaled Mohamed Anwar Aboshanab, Mohammad Mabrouk Aboulwafa, Nadia Abdel-Haleem Hassouna

**Affiliations:** Department of Microbiology and Immunology, Faculty of Pharmacy, Ain Shams University, Organization of African Unity Street, P.O. Box 11566, Abbassia, Cairo, Egypt

## Abstract

Acyl homoserine lactones (AHLs) are the most common class of quorum sensing signal molecules (autoinducers) that have been reported to be essential for virulence of many relevant pathogenic bacteria such as *Pseudomonas aeruginosa*. New approach for controlling infections of such bacteria is through quorum quenching. In this study, the acyl homoserine lactone inhibitory activity of the crude enzyme from a *Bacillus weihenstephanensis*-isolate P65 was characterized. The crude enzyme was found to have relatively high thermal stability and was stable in pH range 6 to 9. The crude enzyme extract was found to have lactonase activity of 36.3 U/mg total protein. Maximum enzyme activity was achieved within a range of 28–50°C and pH 6–9. None of the metals used enhanced the activity neither did EDTA inhibit it. However, a concentration of 10 mM Fe^+2^ reduced the activity to 73.8%. Catalytic activity and kinetic constants were determined using hexanoyl homoserine lactone as a substrate. Studying enzyme substrate specificity using synthetic standard signals displayed broad spectrum of activity. The enzyme was found to be constitutive. Isolation and complete nucleotide sequence of the respective lactonase gene were done and submitted to the Genbank database under accession code KC823046.

## 1. Introduction

 Bacteria communicate with each other using a mechanism known as quorum sensing, a mechanism that is dependent on their population density and which was first reported in the bioluminescent bacterium *Vibrio fischeri *[1].Quorum-sensing bacteria can release, detect, and respond to small signal molecules—the autoinducers (AIs)—that accumulate in the environment as the population grows [2, 3]. At some threshold concentration, the autoinducer effects a change in the gene expression of the population by reentering the cell causing a signaling cascade that ultimately leads to transcriptional regulation of target genes [4]. Quorum-sensing systems have been found in pathogenic bacteria of plants, animals, and humans [5]. Acyl homoserine lactones (AHLs) are the most common autoinducers used by Gram-negative bacteria. The majority of natural AHLs reported to date share conserved structural characteristics, a homoserine lactone ring unsubstituted at the *β*- and **γ**-positions, which is N-acylated at the *α*-position with an acyl group derived from fatty acid biosynthesis (a fatty acyl group) [6]. AHLs vary in their acyl chain lengths (4 to 14 carbons) and/or contain an oxo or a hydroxyl substituent in the acyl chain [2, 3]. Many of the infection-related phenotypes associated with quorum sensing are controlled by AHL/LuxR/LuxI systems: LuxR is the AHL receptor, and LuxI is the AHL synthase. The quorum-sensing systems can induce antibiotic production [7]; pigmentation [8]; biofilm formation [5]; and the expression of virulence factors, for example, proteases, lytic enzymes, exotoxins, rhamnolipids, and extracellular polysaccharides [8, 9]. 

 For example, the opportunistic pathogen *Pseudomonas aeruginosa* possesses two well identified N-acyl homoserine lactone quorum-sensing systems that regulate large, overlapping sets of genes [10]. These 2 distinct *lux *type quorum-sensing systems are termed *las *and *rhl, *which are named after their influence on elastase and rhamnolipid production, respectively. The *las *system regulates the *rhl *system as part of a cascade of virulence regulators. Thus, LasR represents a central checkpoint with the highest degree of interconnection in the network [10, 11]. This hierarchical circuitry controls multiple virulence traits [12]. *Pseudomonas aeruginosa* is also known for its high antibiotic resistance which is attributable to a concerted action of multidrug efflux pumps with chromosomally encoded antibiotic resistance genes and the low permeability of the bacterial cellular envelopes [13]. From here, the targeting of quorum-sensing system represents a novel and promising means of infection control. The idea lies in the development of a drug that attenuates bacterial virulence rather than antibiotic mediated bacteria killing or growth inhibition such that the organism fails to establish successful infection. Compounds with such abilities are termed antipathogenic drugs [14]. 

 An obvious strategy to achieve this is to screen for enzymes capable of degradation of AHL signal molecules [15]. A search for enzymes degrading the AIs of QS systems is promising for designing agents to effectively suppress bacterial infections [16]. Many different bacteria belonging to various genera have been reported to express activity degrading AHLs. 

**Figure 1:**
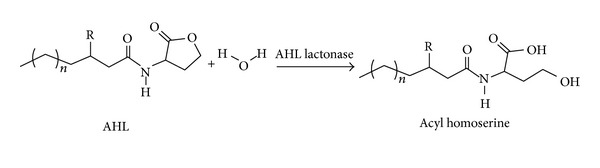
AHL lactonases hydrolyze the lactone ring in the homoserine moiety of AHLs, without affecting the rest of the signal molecule structure [21].

One of the first described and the best characterized enzymes was the AHL-lactonase AiiA24B1, the product of the *aiiA *gene from *Bacillus *sp. 24B1 [17]. Since then, homologues of AiiA lactonase have been discovered in many bacteria belonging to the *Bacillus *genus. All of them show high nucleotide sequence similarity, greater than 90% [18–20]. Lactonases hydrolyze the lactone ring opening rendering the signal molecule inactive, Figure 1. 

In this study, crude lactonase enzyme was collected from a previously identified *Bacillus weihenstephanensis* isolate P65 (Genbank accession code = KC899665) that was isolated from soil. This bacterium was previously screened for its quorum quenching activity against different synthetic AHLs and also against naturally produced AHLs in the extracts of 7 clinically isolated *P. aeruginosa *isolates. The aim of the present was to carry further studies on the enzyme of interest, visualize its stability, characterize its catalytic activity, and substrate specificity, and determine the full enzyme sequence and putative tertiary structure in order to use this enzyme as an antipathogenic drug. 

## 2. Materials and Methods

### 2.1. Chemicals

All chemicals were of high quality from available grades purchased from El-Nasr Chemicals (Adwic), Egypt. Acyl homoserine lactone standards (butanoyl (C4), hexanoyl (C6), heptanoyl (C7), and octanoyl (C8) homoserine lactone) were purchased from Sigma-Aldrich, Germany. Reagents for DNA extraction and PCR were a product of Fermentas, USA.

### 2.2. Bacterial Strains

#### 2.2.1. *Chromobacterium violaceum* CV026

CV026 is mutant strain of *Chromobacterium violaceum* that acts as an acyl homoserine lactone (AHL) dependent biosensor, producing the characteristic purple pigment violacein in response to the presence of the AHL [23]. It was subcultured in a medium containing 20 *μ*g/mL kanamycin for purification as it is kanamycin resistant [24]. It could also be grown without kanamycin. CV026 was subcultured in Luria Bertani (LB) broth for maintenance and stored in slant medium or in lyophilized form for long term preservation.

#### 2.2.2. *Bacillus weihenstephanensis*

It was isolated on R_2_A agar from a soil sample in Cairo, Egypt, and maintained on nutrient agar slants. It was previously screened for its quorum quenching activity and the whole culture activity was characterized. It was identified using 16S ribosomal RNA and was submitted to Genbank under accession code KC899665.

### 2.3. Collection of Crude Enzyme by Sonication

 The *Bacillus* isolate was grown for 16–18 hours in 5 mL LB broth at 28°C with shaking at 160 rpm; then the cells were collected by centrifugation and washed twice with cell washing buffer (Tris-HCl pH 7.5, 25.0 mM). Then, the cells were resuspended in cracking buffer (Tris-HCl pH 7.5, 25.0 mM and Dithiothreitol (DTT) 1.0 mM) and disrupted by 7 minutes of intermittent sonication carried out in ice bath. After sonication, centrifugation was done at 15000 rpm for 15 minutes to remove the unlysed cells and cell debris [25] and the crude enzyme extract was collected. It should be noted that sonication was also done in saline to allow for pH adjustments to test the stability of the enzyme at different pH values.

### 2.4. Measuring the Total Protein Concentration in the Crude Extract

Protein concentration was measured by the method of Lowry et al. [26] using bovine serum albumin as a standard. A standard curve of absorbance at 660 nm as a function of protein concentration of the standard solutions was plotted and used to determine protein concentrations of the sample. The protein concentration was adjusted to 3.5 mg/mL before carrying out further studies. 

### 2.5. Physical Parameters That Affect the Enzyme Stability

#### 2.5.1. Measuring the Thermal Stability

In this assay, 1 mL of the crude enzyme extract (containing about 127 enzyme units) was incubated at 50, 80, and 90°C for 30 and 60 minutes. Then, 90 *μ*L of each aliquot was pipetted into the wells of semisolid LB agar to measure the activity using well diffusion method described by Ravn and coworkers [24]. The semisolid seeded agar was prepared as follows: a preculture of CV026 was grown overnight in 5 mL LB broth at 28°C with shaking at 160 rpm and then transferred to 100 mL molten semisolid agar (1.2% w/v) maintained at 46°C. 1 mL of 10 *μ*M synthetic hexanoyl homoserine lactone (HHL) was also transferred to the semisolid agar. After that, 10 mL of the semisolid agar was poured over the surface of prewarmed LB agar plates. When the overlaid agar had solidified, wells were punched with a sterile Cork borer (diameter 10 mm). The plates were then incubated at 28°C for 24 hours. The growth of CV026 showed purple color in the whole plate except the zones around the wells and the residual activity was measured by comparing the inhibition zone diameters of the test to that of the control. The experiment was done in three replicates and the mean deviation and standard deviation were calculated.

#### 2.5.2. Measuring the Stability at Different pH

 The assay was carried out according to Cao and coworkers [27] with modifications as follows: 1 mL aliquots of the crude enzyme extract (containing about 127 enzyme units) were transferred to Wasserman tubes and the pH of each was changed to the desired pH using either McIlvaine buffer (prepared by mixing 0.4 mL 0.2 M Na_2_HPO_4_ and 19.6 mL 0.1 M citric acid) or Glycine-NaOH buffer (0.1 M Glycine-NaOH adjusted to pH 12). The tubes were incubated at room temperature for 30 minutes and then neutralized to pH 7 with the counter buffer. Afterwards, the volumes of all the aliquots were adjusted equally using saline. Then, 90 *μ*L of each aliquot was pipetted into wells formed in LB agar seeded with cv026 and supplemented with 100 nM HHL for the development of inhibition zones. Control was done using 90 *μ*L of the crude extract that was incubated at room temperature for the same time and had its volume adjusted with saline to be equal to that of test. The experiment was done in three replicates and the mean deviation and standard deviation were calculated. The stability of the enzyme was studied over a pH range of 4 to 10.

### 2.6. The Effect of Some Divalent Metals and EDTA on Activity

Concentrations of 1 mM and 10 mM of Fe^++^, Mg^++^, Ca^++^, and EDTA were added to the crude extract and incubated for 30 minutes. For Zn^++^ and Cu^++^, a concentration of 10 mM was found to have an inhibitory activity on cv026 growth so they were used in a concentration of 1 and 2 mM. A sample without an additive served as a control. Then, 90 *μ*L of the control and the tested aliquots were pipetted into the wells of semisolid LB agar as previously described and the inhibition zone diameters were compared to that of the control. The experiment was done in three replicates and the mean deviation and standard deviation were calculated.

### 2.7. The Effect of Adding Signal Molecule to the Growth Medium

The isolate was grown in 5 mL LB containing 1 mM HSL then sonication was done, and the well diffusion assay was carried out as previously described to measure the zone diameters and compare them to the control grown without a signal in the growth medium. 

### 2.8. Measuring the Catalytic Activity

This was done according to Cao and coworkers [27] with modifications as follows: aliquots of crude enzyme extract (0.5 mL) were incubated with 10 *μ*M HHL at 28°C with shaking at 160 rpm. Then, the reaction was terminated by adding 2% SDS. This was done at 1.5, 3, 5, and 7 hours. A control was done using plain buffer containing 10 *μ*M HSL incubated under the same conditions for the same time and also treated with SDS. After termination of the reaction, the residual amount of HHL was measured by well diffusion assay as follows: 10 mL LB agar were overlaid by 10 mL semisolid agar seeded with cv026 and then left to solidify. Wells were punctured into the agar and 60 *μ*L of the tested samples were transferred into them. The plates were then incubated at 28°C for 24 hours for color development. The homoserine lactones (HSL) in the wells formed zones of purple color around the wells, the diameter of which was proportional to the concentration of HSL. The measured zones were then compared to standard curve showing the diameters of the purple zones formed as a function of the different concentrations of HHL (with 2% SDS) pipetted into the wells. One unit of AHL lactonase activity was defined as the amount of enzyme that hydrolyzed 1 nM HHL per minute. 

#### 2.8.1. Effect of Temperature on Catalytic Activity

This was done as described in the determination of the catalytic activity of the crude enzyme but with the incubation temperature (of the crude extract with HSL) changed once to 5, 20, 40, 50, and 70°C. A control was prepared and incubated at 28°C; then the initial rate of reaction was determined after 1.5 h for both the control and the test.

#### 2.8.2. Effect of pH on Catalytic Activity

Aliquots of crude enzyme extract used in this assay, each of about 1 mL volume (containing about 127 enzyme units), had their pH values adjusted to 6, 7, 8, and 9 using McIlvaine and glycine-NaOH buffer. Then, they were incubated with HSL of concentration 10 *μ*M at 28°C. After 1.5 h the reaction was stopped using SDS 2% and 60 *μ*L was pipetted into wells of semisolid LB seeded with cv026 overlaying LB agar as previously described. The plates were then incubated at 28°C for 24 hours and the diameters of the formed zones were measured and compared to standard curve to determine the residual amount of HHL. The activity at different pH was compared to the activity at pH 7.

### 2.9. Determination of Kinetic Constants

 Aliquots of crude enzyme extract (0.5 mL containing 63.5 enzyme units) were incubated with different concentrations of HSL: 5, 10, 15, and 20 *μ*M at 28°C with shaking at 160 rpm. Then, the reaction was terminated by adding 2% SDS after 90 minutes and the residual amount of HHL was measured by well diffusion assay. The initial velocity of the reaction was calculated from the following equation:

(1) $$\begin{array}{c} \text{rate}\;\;\text{of}\;\;\text{AHL}\;\;\text{degradation}\;=\frac{-d\left[A\right]}{d\left[t\right]}, \end{array}$$

where *A* is the concentration of AHL and *t* is the time in minutes. The data were plotted as a Lineweaver-Burk curve also called the double reciprocal curve. Afterwards, kinetic constants for the crude enzyme extract, *V*_max⁡_, *K*_cat_, and *K*_*m*_, were calculated. *V*_max⁡_ represents the maximum velocity of reaction, *K*_cat_ represents the turnover number and was calculated relevantly to the total protein concentration (1 mg protein is equivalent to 36.3 lactonase units), and *K*_*m*_ represents Michaelis-Menten constant which is the concentration of the substrate at half *V*_max⁡_. 

### 2.10. Enzyme Substrate Specificity

The ability of the enzyme to hydrolyze different acyl homoserine lactones was assessed using standard signals, butanoyl (C4), heptanoyl (C7), and octanoyl (C8) homoserine lactones. This was done by measuring the catalytic activity of the enzyme as described above for hexanoyl homoserine lactone. The reaction was carried out for 90 min at 28°C and pH 7. Units of lactonase activity to total protein concentration was measured in terms of C4, C7, and C8 HSL and compared to that measured previously using HHL standard.

### 2.11. Chromosomal DNA Extraction

 It was done according to Pospiech and Neumann [28], and finally, the extracted DNA was dissolved in 500 *μ*L TE buffer containing RNase with a concentration of 100 *μ*g/mL and placed in eppendorf tubes as aliquots of 20 *μ*L each, stored at −20°C to be used as a template for the amplification of lactonase gene.

### 2.12. Amplification of Lactonase Gene

Primers were designed using data that were collected from NCBI GenBank (http://www.ncbi.nlm.nih.gov/), which were then used to design the suitable primers using ClustalW (http://www.genome.jp/tools/clustalw/) program. The primers were afterwards checked for their specificity to the required gene using Primer 3 program (http://primer3.sourceforge.net/). 

The 2 primers used were as follows: PHlact-F: 5′ ATGACAGTAAAGAAGCTTTATTTCG 3′ and PHlact-R: 5′ CTATATATACTCTGGGAACACTTTAC 3′. The PCR was done according to the following thermal cycling conditions: initial denaturation was carried out at 95°C for 5 min. Then, 30 cycles of denaturation (at 95°C for 30 sec), annealing (at 50°C for 50 seconds), and extension (at 72°C for 1 min) were carried out. Final extension took place at 72°C for 5 min.

### 2.13. Agarose Gel Electrophoresis

Agarose gel electrophoresis was carried out as described by Sambrook and Russell using 0.8% agarose gel containing 0.1 *μ*g/mL ethidium bromide [29].

### 2.14. DNA Sequencing and Accession Code of Lactonase Gene

Sequencing of PCR product was done by GATC Company through Sigma scientific company, Egypt. It was done by the use of ABI 3730xl DNA Sequencer. The obtained sequence was analyzed, and assembled using Staden package program version 3 [30]. The ORF was detected using FramePlot [31] and the full nucleotide sequence was submitted to GenBank database under the accession code KC823046.

### 2.15. Prediction of the Tertiary Structure of the Lactonase Enzyme

The putative tertiary structure of the lactonase enzyme was predicted using Swiss-Model software (http://swissmodel.expasy.org/) [32–34]. This was done to visualize the predicted conformation of the protein and the possible metal-binding residues which might have an effect on the enzyme activity.

## 3. Results 

### 3.1. Physical Parameters That Affect the Enzyme Stability

#### 3.1.1. Thermal Stability of the Enzyme

**Figure 2:**
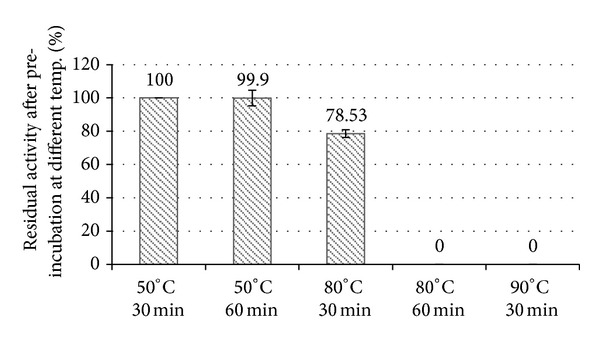
Residual activity of the enzyme after preincubation at different temperatures.

 The crude enzyme retained >90% of its activity when incubated at 50°C for 30 and 60 minutes. When incubated at 80°C for 30 minutes, it retained about 78.5% of the activity. However, when incubated at 80°C for 60 minutes, the activity was lost completely. The same happened when the crude enzyme was incubated at 90°C for 30 minutes where the activity was completely abolished evidenced by the absence of any inhibition zones in comparison to the control. The results represented in Figure 2 show the residual activity of the enzymeafter incubation at different temperatures from which the enzyme is found to have relatively high thermal stability. 

#### 3.1.2. The Stability at Different pH

**Figure 3:**
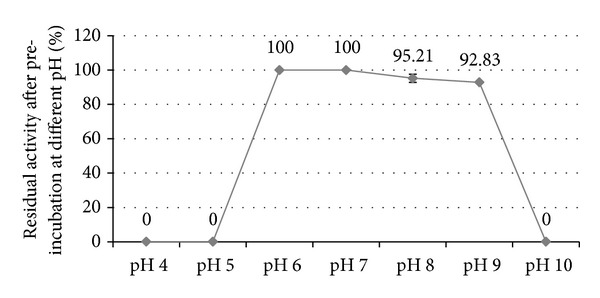
Residual activity of the enzyme after preincubation at different pH.

As shown from the results in Figure 3, the isolate retained >90% of the activity when preincubated at pH 6, pH 8, and pH 9 compared to the control preincubated at pH 7 while the activity was lost completely after preincubation at pH 4, pH 5, and pH 10. 

#### 3.1.3. Effect of Some Divalent Metals and EDTA on Activity

**Figure 4:**
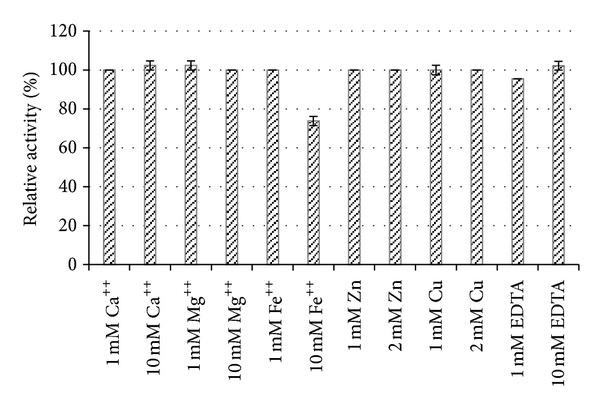
Effect of metal ions on activity.

At all the concentrations of Ca^++^, Mg^++^, Zn^++^, and Cu^++^ and also EDTA used in the assay, the enzyme retained ~100% of its activity. The activity was neither stimulated nor inhibited. It also retained ~100% of the activity at a concentrated 1 mM of Fe^++^ while at a concentration of 10 mM, the activity was reduced to 73.8%. Results are shown in Figure 4. 

#### 3.1.4. The Effect of Adding Signal Molecule to the Growth Medium

Relative activity of the crude enzyme of the isolate grown in LB containing the HSL was found to be equal to 98.86% ± 1.14 of the activity of the crude enzyme of isolate grown without HSL in the medium. Activity which came almost equal indicates that the AHL degrading lactonase is a constitutive enzyme and its productivity is not enhanced by adding the substrate to the growth medium.

#### 3.1.5. Measuring the Catalytic Activity of the Crude Enzyme

**Figure 5:**
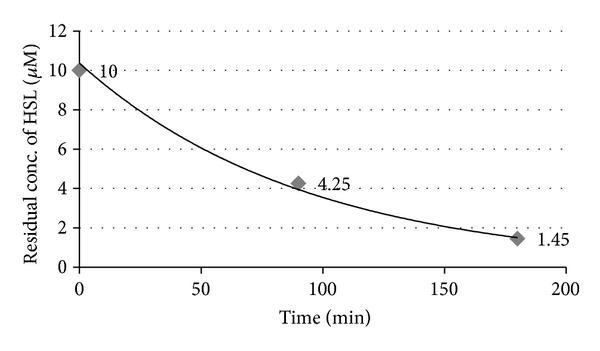
Catalytic rate of reaction using HHL as a substrate.

As shown in Figure 5, the initial rate of reaction (during the first 90 minutes) was 36.28 nM·min^−1^ mg^−1^ and then it dropped to 17.77 nM·min^−1^ mg^−1^. At 5 hours, no zone was formed indicating the complete degradation of HHL. According to the initial reaction velocity, the activity in terms of AHL lactonase activity to the total protein concentration was found to be equal to 36.3 U/mg total protein.

#### 3.1.6. Effect of Temperature on Catalytic Activity

**Figure 6:**
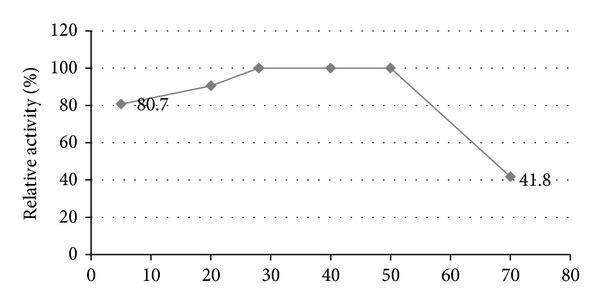
Effect of temperature on catalytic activity.

Results, Figure 6, show that the activity was almost not affected between 28 and 50°C. At 20°C, activity was reduced to 90.5% and to 80.7% at 5°C. However, it was greatly affected at 70°C and the activity dropped to about 41.8% of the maximum activity. 

#### 3.1.7. Effect of pH on Catalytic Activity

**Figure 7:**
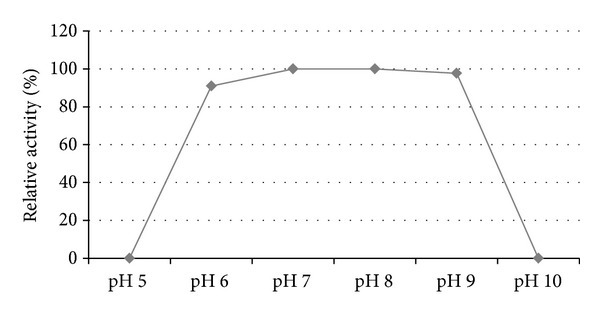
Effect of pH on catalytic activity.

Result, Figure 7, showed that activity was almost unchanged over the studied pH range where the enzyme retained >90% of maximum activity between pH 6 and pH 9. The AHL Lactonase activity to the total protein concentration described as lactonase units/mg total protein remained almost unchanged within this range. The activity at pH values outside this range was not assessed as the enzyme proved to be unstable at pH 5 and pH 10 as concluded earlier. 

### 3.2. Determination of Kinetic Constants

Maximum rate of reaction (*V*_max⁡_) was found to be equal to 460.5 nM·min^−1^, *K*_cat_ was equal to 52.4 min^−1^, and Michaelis-Menten constant (*K*_*m*_) was found to be equal to 77.13 nM. 

### 3.3. Enzyme Substrate Specificity

By quantification of the residual AHL, we determined the relative enzyme activity of AHL-lactonase on different AHL derivatives. The data indicate that AHL lactonase has a broad substrate spectrum and digests efficiently all of the 4 tested AHL compounds used in the study, Table 1. One unit of lactonase activity was defined as the amount of enzyme that hydrolyzed one nM of the AHL tested in 1 min. Within a narrow range of difference in activity, maximum activity was observed against HHL (C6-HSL) as a substrate with slight difference when compared to activity against C4-HSL and C7-HSL while least degrading activity was observed against C8-HSL. 

### 3.4. Lactonase Gene Amplification

 Using the extracted chromosomal DNA, an expected PCR product (the amplified gene) of 0.75 Kb was detected using agarose gel electrophoresis.

### 3.5. Full Lactonase Gene/Enzyme Sequence

**Figure 8:**
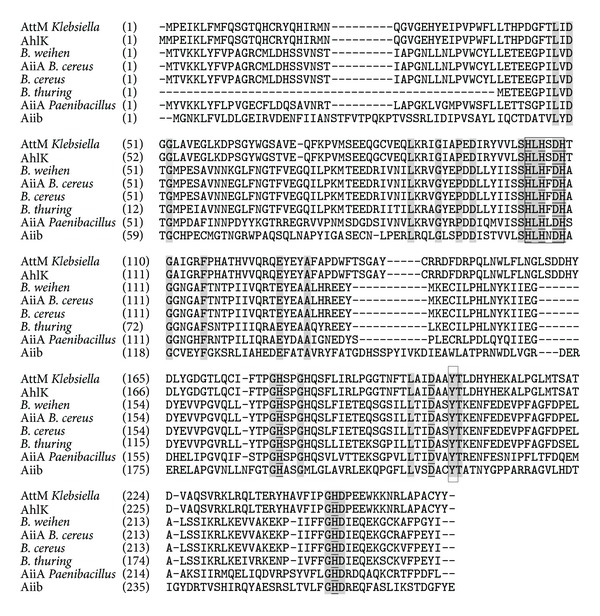
Alignment of the amino acid sequence of lactonase from *B. weihenstephanensis* (accession = KC823046) with other homologs using ClustalW. Lactonase was aligned with AttM lactonase of *Klebsiella pneumoniae *VA360 (accession code = Zp-23552218), AhlK of *Klebsiella pneumonia* (accession = AAO47340.1), AiiA of *Bacillus cereus* (accession code = AAY22194), metallo-beta-lactamase protein from *Bacillus cereus* (accession = ZP_03103014.1), lactonase of *Bacillus thuringiensis* serovar sotto (accession = ZP_04127391.1), AiiA of *Paenibacillus alvei* DSM 29 (accession = ZP_10863318.1), and Aiib from *Agrobacterium tumefaciens* (accession: 2R2D_E). H*X*H*X*DH motif and Tyr (194) were boxed, identical residues were shaded with grey, and the proposed metal ligands according to Thomas and coworkers [22] for the dinuclear zinc form of AHL lactonase were underlined.

Sequencing revealed the presence of the motif HXHXDH. The presence of a tyrosine (Y) residue at the position 194 was also observed in our sequence. Using NCBI database and ClustalW, amino acid sequence alignment of our enzyme with AHL lactonases from other species was done and is displayed in Figure 8. 

### 3.6. Prediction of the Tertiary Structure of Lactonase Enzyme

**Figure 9:**
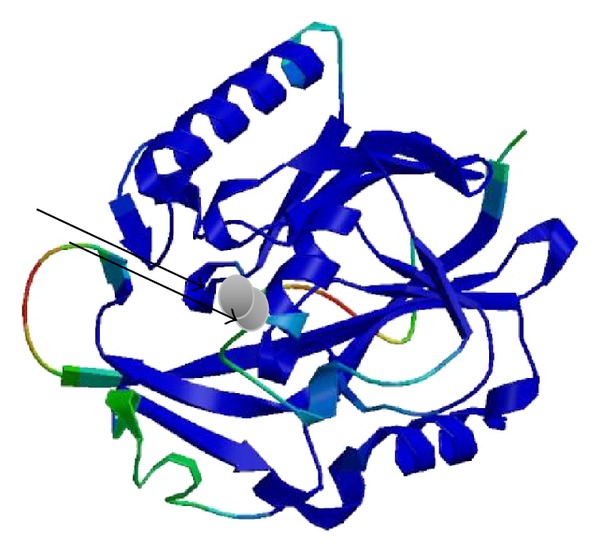
The putative tertiary structure of lactonase enzyme of *B. weihenstephanensis*, accession code Genbank KC823046. The two arrows show the suggested metal ligands for dinuclear zinc binding.

The putative tertiary structure of lactonase gene, shown in Figure 9, was predicted by Swiss-Model software using N-acyl homoserine lactone hydrolase of *Bacillus thuringiensis* serovar kurstaki as a template (accession: 3DHA_A). The model showed a QMEAN4 score of 0.862 [35]. The model displays that our lactonase enzyme has a putative structure of a metalloenzyme that is hypothesized to contain binding sites for 2 zinc atoms where all the residues interacting with the ligand are completely conserved between model and template. 

## 4. Discussion

 Here, we studied the activity of crude AHL degrading enzyme from the *B. weihenstephanensis *which was found to be closely related to lactonases that are widespread in *B. thuringiensis *and *Bacillus cereus *strains. AHL lactonases have been used previously to demonstrate that quenching bacterial quorum sensing is a promising strategy for preventing and controlling bacterial infections. The data presented here show that the AHL lactonase encoded by *B. weihenstephanensis *is a good candidate for further development into an effective drug for infection control. 

 From results in this study, the enzyme proved to have high thermal stability retaining >78% of its activity after 30 min of preincubation at 80°C. In 2012, Cao and coworkers studied the thermal stability of a lactonase enzyme and stated that it retained about 60% of its activity after incubation for 20 min at 80°C or for 3 min at 90°C [27]. Another study on a recombinant AHL-lactonase stated that the enzyme exhibited thermal stability at 70°C, retaining more than 80% of the initial activity after preincubation at 70°C for 30 min [36]. A third study also showed that AHL-lactonase exhibited excellent thermal stability at temperatures below 37°C, and the purified enzyme, kept at 4 and 21°C for 10 days, still maintained about 99% activity. But the enzyme was less stable at higher temperatures; its activity was decreased sharply after incubation for 2 h at 45°C [37]. Another study on a lactonase enzyme isolated from the archaeon organism *Sulfolobus islandicus* showed that the enzyme exhibited respective half-lives of 84 ± 20 min, 8.5 ± 1.5 min, and 3.6 ± 0.4 min at 85, 90, and 95°C [38]. From the previous findings, our enzyme of interest here is believed to establish relatively excellent stability at high temperatures.

 Several studies reported that pH has a drastic effect on the conformational structure of AHL-lactonase. In 2004, Wang and coworkers stated that lactonase enzyme was unstable at low pH where, according to this study, the asymmetric conformational structure of AHL-lactonase remained unchanged in pH ranging from 7 to 9 and slightly changed at pH 6 but significantly changed at pH 5.5 and completely lost at pH 5 [37]. Two previous studies also found that lactonase enzyme was unstable below pH 6 [27, 36]. In the same two studies, the protein was stated to be stable between pH 6–11 and pH 6–12 (maintaining >70% of its activity), respectively [27, 36]. In our present study, the enzyme displayed high stability over a pH range of 6–9 retaining about >90% of its activity. However, it was found to be unstable at pH 10 evidenced by loss of activity after preincubation at pH 10 for 30 min. 

 Our study here supports the idea that lactonase activity is not enhanced by the presence of divalent metal ions nor it is inhibited by the presence of metal chelators. The activity was partially inhibited by high concentration of Fe^++^. Consistent with the results in our study are the findings stated by Cao and coworkers [27] who stated that none of the metal ions used in that study enhanced the activity while a concentration of 10 mM Fe^++^ partially inhibited the activity. In 2004, Wang tested several metal ions, including Mg^++^, Ca^++^, Mn^++^, Co^++^, Zn^++^, Cd^++^, and Ni^++^ which showed no effect on enzyme activity at 0.2 and 2 mM concentrations. On the other hand, AHL-lactonase was partially inhibited by Cr^++^, Fe^++^, and Pb^++^ and completely inhibited by Cu^++^ [37]. The study stated that this was possibly due to reaction with sulfhydryl groups of the enzyme. Another study also reported the inhibitory activity of Cu^++^ on lactonase enzyme [36]. In our study here, however, Cu^++^ showed no inhibitory activity on the lactonase enzyme. Another study carried out in 2010 [36] showed that activity of lactonase was enhanced upon the addition of 1 mM and 10 mM Ca^++^ to 106% and 117.5%, respectively, and also upon the addition of 10 mM Mg^++^ the activity increased to 108%. It is clear, therefore, that the effect of different metals ions on activity of lactonase is still controversial. Several studies stated that lactonase is a phosphodiesterase-like metalloprotein and clarified the importance of divalent metal ions for its activity [22, 38, 39]. Other studies, like the one carried out by Wang and coworkers, stated that although trace amounts of Zn^++^ were measured in the enzyme, the AHL lactonase is not a metalloenzyme [37]. In our study here, EDTA used in concentrations of 1 and 10 mM as a divalent metal ion chelator showed no effect on activity emphasizing that divalent metal ion presence does not seem to be essential for activity. According to Wang and coworkers [37], both removal of Zn^++^ from AHL-lactonase by the ion-chelating reagent EDTA and its addition did not affect enzyme activity and hence they stated that the HXHXDH sequence seems to serve as a zinc-binding site in some enzymes but not in others and concluded that AHL-lactonase does not belong to metallohydrolase family. 

 Studying the catalytic activity, the crude enzyme could effectively degrade 10 *μ*M in 5 hours. The AHL lactonase activity of the crude extract with total protein concentration of 3.5 mg/mL was found to be equal to 36.3 U/mg total protein. In a study on a recombinant lactonase in 2012, Cao and coworkers found that an AHL lactonase activity of 2.5 U/mL was measured in the cell lysate at the end of culture period using 3-oxo-C8-HSL as a substrate as compared to 127 U/mL in our study. When purified, the activity increased to about 8.54 U/mL, more than threefold [27]. Accordingly, these findings emphasize that the enzyme in our study has high potential to be developed into an antipathogenic drug of high activity. 

 Catalytic activity was not affected by temperature within a range of 28–50°C displaying maximum activity in that range. Below 28°C, activity was slightly reduced. At 70°C, the enzyme activity dropped to less than 50%. According to a study carried out by Wang and coworkers, lactonase enzyme inactivation was noticed at 45°C [37]. 

 Several previous studies displaying the effect of pH on activity were carried out. A purified recombinant lactonase (AiiAB546) had the optimum pH of 8.0 and retained more than 73% of the maximum activity at pH 6.5–8.9 [36]. Another enzyme (AiiAAI96) retained about 95% of the maximal activity between pH 6.0 and 8.5 at 30°C [27]. In another study that determined the effect of pH on AHL-lactonase activity in a range from pH 5 to pH 9 using 3-oxo-C8-HSL as a substrate, AHL-lactonase activity, enhanced with pH increasing from 6 to 8, reached the maximum at pH 8 and then declined slightly at pH 9 [37]. In our study here the enzyme activity remained almost unchanged within pH 6–9 maintaining over 90% of its maximum activity over that range.

 The broad spectrum of the enzyme activity was displayed by its ability to degrade all the tested AHL synthetic standards. It is also worth mentioning here that the enzyme previously showed high activity against the naturally produced signals in the extracts of 7 clinically isolated *P. aeruginosa* during the screening process as mentioned earlier. Several studies support that lactonase enzyme has a nonspecific substrate activity against homoserine lactones [27, 37]. Although lactonase activity is believed somewhat to be affected by the length of the acyl chain of the substrates [37], our study here showed that highest activity was displayed against C6-HSL followed by C7-HSL then C4-HSL with very slight differences between them. Activity on C8-HSL came last but also within a narrow window of variation. In 2004, Wang and coworkers also reported that the best substrates of AHL-lactonase are C6-HSL among the reduced AHL molecules and 3-oxo-C10-HSL of the C3 substituted AHL signals [37].

 Amplification and sequencing of lactonase gene were done for the sake of full data collection about the enzyme to allow for further studies. Sequence analysis revealed the presence of several highly conserved domains shared with lactonases from other species. It revealed the presence of the motif H*X*H*X*DH which is believed to play a role in metal binding in metalloproteins and is conserved among almost all metalloenzymes. Thomas and coworkers reported that comparison of AHL lactonase with other superfamily members including glyoxalase II, phosphodiesterase-ZiPD, and methyl parathion hydrolase suggests possible zinc binding residues which are totally conserved in all known AHL lactonases: His104, His106, and His169 for the metal-1 site and Asp108, His109, and His235 for the metal-2 site [22]. The presence of a tyrosine (Y) residue at the position 194 was also observed in our sequence. According to Elias and coworkers, this tyrosine residue is believed to be conserved in all lactonase sequences and is believed to play a role in the positioning of lactone ring of the substrate [40]. The study also reports that lactonase is a metalloenzymes. However, Wang and coworkers stated that although sequence alignment of AHL-lactonase with these metallohydrolases revealed that they share this consensus motif known as H*X*H*X*DH at the central region, AHL-lactonase does not belong to metallohydrolase family [37]. In our study here, the presence of H*X*H*X*DH motif, residues proposed to be Zn^++^ binding sites and the conserved tyrosine residue, was reported as displayed earlier. Besides, the putative tertiary structure of our enzyme also suggested the presence of 2 zinc binding metal sites. However, whether these sites are truly metal-binding residues and whether lactonase is a metalloenzyme still need to be further examined.

## 5. Conclusion

 AHL lactonase in this study appears to be a potent enzyme, demonstrating excellent thermal stability and retaining maximum activity over the studied pH range. It also has strong catalytic activity and a broad spectrum of activity against AHL signal molecules. Other than partial reduction in activity by 10 mM Fe^++^, lactonase enzyme in this study proved to be resistant to the effect of heavy metals and metal-chelating reagent, EDTA. Unlike other lactonases, the enzyme activity is not inhibited by the presence of copper. The enzyme contains the conserved H*X*H*X*DH sequence resembling the zinc binding motif of several groups of metallohydrolase family and shares other sequences highly conserved among lactonases from other species. Putative tertiary structure of the enzyme also suggests the presence of binding sites for two zinc atoms. Further studies on this enzyme are recommended as it represents a new outstanding tool for quorum quenching and, consequently, virulence suppression and infection control.

## Figures and Tables

**Table 1:** Hydrolytic activity on different AHL standards.

Substrate	Lactonase activity units/mg total protein
C4-HSL	30.16 U/mg
C6-HSL	36.3 U/mg
C7-HSL	32.02 U/mg
C8-HSL	21.2 U/mg
